# Race disparity in blood sphingolipidomics associated with lupus cardiovascular comorbidity

**DOI:** 10.1371/journal.pone.0224496

**Published:** 2019-11-20

**Authors:** Samar M. Hammad, Jasmyn R. Hardin, Dulaney A. Wilson, Waleed O. Twal, Paul J. Nietert, James C. Oates

**Affiliations:** 1 Department of Regenerative Medicine & Cell Biology, Medical University of South Carolina, Charleston, South Carolina, United States of America; 2 College of Graduate Studies/Summer Undergraduate Research Program, Medical University of South Carolina, Charleston, South Carolina, United States of America; 3 Department of Public Health Sciences, Medical University of South Carolina, Charleston, South Carolina, United States of America; 4 Department of Medicine, Division of Rheumatology & Immunology, Medical University of South Carolina, Charleston, South Carolina, United States of America; Oklahoma Medical Research Foundation, UNITED STATES

## Abstract

Systemic lupus erythematous (SLE) is a chronic multi-organ autoimmune disease. Genetic and environmental factors contribute to disease onset and severity. Sphingolipids are signaling molecules involved in regulating cell functions and have been associated with multiple genetic disease processes. African-Americans are more likely to suffer from SLE morbidity than Whites. The Medical University of South Carolina has banked plasma samples from a well-characterized lupus cohort that includes African-Americans and Whites. This study examined the influence of race on plasma sphingolipid profiles in SLE patients and association of sphingolipid levels with comorbid atherosclerosis and SLE disease activity. Mass spectrometry revealed that healthy African-Americans had higher sphingomyelin levels and lower lactosylcermide levels compared to healthy Whites. SLE patients, irrespective of race, had higher levels of ceramides, and sphingoid bases (sphingosine and dihydrosphingosine) and their phosphates compared to healthy subjects. Compared to African-American controls, African-American SLE patients had higher levels of ceramides, hexosylceramides, sphingosine and dihydrosphingosine 1-phosphate. Compared to White controls, White SLE patients exhibited higher levels of sphingoid bases and their phosphates, but lower ratios of C16:0 ceramide/sphingosine 1-phosphate and C24:1 ceramide/sphingosine 1-phosphate. White SLE patients with atherosclerosis exhibited lower levels of sphingoid bases compared to White SLE patients without atherosclerosis. In contrast, African-American SLE patients with atherosclerosis had higher levels of sphingoid bases and sphingomyelins compared to African-American SLE patients without atherosclerosis. Compared to White SLE patients with atherosclerosis, African-American SLE patients with atherosclerosis had higher levels of select sphingolipids. Plasma levels of sphingosine, C16:0 ceramide/sphingosine 1-phosphate ratio and C24:1 ceramide/sphingosine 1-phosphate ratio significantly correlated with SLEDAI in the African-American but not White SLE patients. The C16:0 ceramide/sphingosine 1-phosphate ratio in SLE patients, and levels of C18:1 and C26:1 lactosylcermides, C20:0 hexosylceramide, and sphingoid bases in SLE patients with atherosclerosis could be dependent on race. Further ethnic studies in SLE cohorts are necessary to verify use of sphingolipidomics as complementary diagnostic tool.

## Introduction

Systemic lupus erythematosus (SLE) is a chronic autoimmune disease with heterogeneous organ involvement and severity. The cause of SLE is unknown, and there is at present no cure. The majority (90%) of people with lupus are females, and African-American women are three times more likely than White women to have lupus and develop severe symptoms [1]. Despite major efforts to phenotype SLE, there is still a need to identify biomarkers that can serve to track or predict disease activity as well as treatment efficacy. Dysregulation of the sphingolipid pathway has been described in several inflammatory and immune-mediated diseases [2, 3]. Recently, alterations in the sphingolipid pathway in SLE and some of its related complications in both humans and murine models have been described [4–8]. However, to date no studies have been conducted to determine whether changes in sphingolipid metabolism in SLE patients may be associated with the development of cardiovascular disease (CVD), and no studies have investigated whether such changes may be impacted by race.

Sphingolipids are both structural lipids and signaling molecules with significant physiologic functions [9]. Sphingolipids are associated with cellular membranes and plasma lipoprotein, and their synthesis and degradation are tightly regulated to maintain homeostasis [9, 10]. The central molecule in sphingolipid metabolism is ceramide (Cer), which is generated by de novo synthesis or through the action of sphingomyelinase (SMase). Cer may be hydrolyzed by ceramidase (CDase) to liberate sphingosine, which can be re-acylated to Cer or phosphorylated to sphingosine 1-phosphate (S1P) by sphingosine kinase (SK). Cellular accumulations of Cer and sphingosine have been associated with apoptosis [9, 11]; whereas, S1P was found to promote endothelial integrity and lymphocyte migration [12]. S1P may also be pro-inflammatory, and serum S1P has been suggested as a biomarker in coronary artery disease [13]. The inhibition of S1P through anti-S1P antibodies (Lpath) and FTY720 (Fingolimod) has been explored as treatment for inflammatory diseases (e.g., multiple sclerosis) [14]; however, the role of S1P in lupus is still to be determined.

The risk for CVD is almost ten times higher in patients with autoimmune diseases than in the general population. Despite the dyslipidemia and accelerated CVD associated with SLE [15], the significance of the conventional plasma lipid panel (e.g., cholesterol and triglycerides) in the diagnosis/prognosis of CVD in SLE patients has been in question. The efficacy of statins to prevent atherosclerosis in SLE was found to be inconsistent [16]. Furthermore, African-Americans normally have lower triglycerides and higher HDL cholesterol levels than other ethnicities; however, paradoxically they have increased risk of CVD [17]. Abnormal sphingolipid metabolism is associated with multiple genetic and metabolic diseases [10]. Sphingolipids in blood are carried on circulating lipoprotein particles (HDL, LDL, and VLDL) and their use as disease biomarkers has been explored [10]. Plasma sphingomyelin (SM) levels have been independently associated with CVD [18] and subclinical atherosclerosis [19]. Lately, a study on a European SLE cohort showed that dysregulations in circulating sphingolipids is associated with renal complications in SLE [20]; however the race effect was not tested. Because African-Americans are more likely to develop more severe symptoms than Whites [1], we sought to identify certain sphingolipid species as potential biomarkers that can predict or indicate SLE disease activity and associated CVD, and may explain at least in part the race disparity in SLE. We at the Medical University of South Carolina (MUSC) have a unique access to a well-characterized lupus population that includes both African-Americans and Whites. In this study, we investigated the extent to which sphingolipids were associated with SLE, with comorbid atherosclerosis, and SLE disease activity. In each of these research goals, we also sought to determine the extent to which the associations were modified by race.

## Materials and methods

### Ethics statement

The study was approved by the ethics committee of the Medical University of South Carolina and was performed according to the protocols approved by our institutions’ institutional review board (IRB), (Protocol number: Pro00021985). Prior to enrollment in the study, written informed consent was obtained from each subject. All data were analyzed anonymously.

### Study subjects

The MUSC Clinical and Community Resource Core contained within MUSC’s Core Center for Clinical Research (CCCR) is designed to recruit, retain, clinically characterize patients with lupus, and to link clinical laboratory and biological sample information in a single database. Since April of 2013, 411 new lupus and 141 control participants have been enrolled. Healthy controls were recruited from the clinic in University population as a convenience sample. Plasma samples have been collected, often across multiple visits, from 358 lupus patients and 179 healthy controls (negative for autoimmune disease by the Connective Tissue Disease Screening Questionnaire (CSQ)) [21]. Subjects are assessed by MUSC rheumatologists, all of whom are trained in SLE criteria and activity measures. For this study, a nested subset of this population of patients with four or more American College of Rheumatology (ACR) criteria for SLE was selected based on ability to match with healthy controls by age (within 5 years), sex, and self-reported race. The nested subset of the SLE registry population shown in S1 Fig reflects the general demographics of the registry population.

Samples were collected and routed for immediate processing with storage in aliquots at -80°C. This core provides ready access to longitudinal information on patient phenotype (e.g., SLE diagnosis by American College of Rheumatology criteria [22, 23], co-variables such as age, medication, disease activity by SLE disease activity index (SELENA-SLEDAI with SLEDAI-2K proteinuria definition) [24] and outcomes such as CVD, and location of visit-specific patient samples. For this study, n = 73 female SLE patients and n = 34 unrelated controls were selected from the cohorts for comparison of their sphingolipid profiles. These sample sizes provided 80% power for detecting differences equivalent to effect sizes of 0.6 between SLE patients and controls and moderate to high correlations (e.g. rho>0.33) between sphingolipid measurements and SLEDAI scores among the SLE cases. The presence of atherosclerotic complications such as stroke, myocardial infarction, angina, coronary bypass, and claudication were determined by history, physical, and chart review by experienced rheumatology clinicians and recorded as elements of the SLE International Cooperating Clinics Damage Index (SLICC-DI).

### Sphingolipid extraction and analysis

Mass spectroscopy was used to measure plasma levels of individual species of five classes of sphingolipids: Cers, sphingoid bases: sphingosine and dihydrosphingosine (dhSph) and their phosphates (S1P and dhSph-1P, respectively), SM, and hexosyl- and lactosylceramides (Hex-Cer and Lact-Cer, respectively) as previously described [25–30]. Briefly, 100 μl of de-identified plasma sample from each subject was spiked with internal standards and the sphingolipid complement in each sample was quantitatively extracted. The sphingolipids in plasma extracts were separated and their masses quantitated using high performance liquid chromatography-tandem mass spectrometry (LC-MS/MS) as described previously [25–30]. Lipids eluted during chromatography were detected and quantitated using a Thermo Scientific Quantum Access triple quadruple mass spectrometer equipped with an electrospray ion source (ESI) operating in multiple reaction monitoring (MRM) positive ion mode. Chromatographic separations were obtained under a gradient elution of a Peeke Scientific (Redwood City, CA), Spectra C8SR 150×3.0 mm; 3-μm particle size column. Quantitative analyses were based on calibration curves generated by injecting known amounts of the target analytes and an equal amount of the internal standards. A listing of the internal standards used, and of the sphingolipids with available calibration standards was previously published [25]. The calibration standards were obtained predominately from the MUSC Lipidomics Share Resource facility, and from commercially available sources, Avanti Polar Lipids Inc. and Matreya LLC. Molecular species of sphingolipids, which do not have available standards were quantified using the calibration curve of the closest eluting counterpart. The final concentrations of analytes in the samples were determined using the appropriate corrections for sample loss based on internal standard recovery calculations. The resulting data was then normalized to the volume of sample analyzed. Final results were reported as pmol/ml plasma.

### Statistical analyses

All data were analyzed anonymously. Demographics and clinical characteristics were summarized for SLE patients and controls, and comparisons between them were conducted using t-tests, chi-square tests, or Fisher’s exact tests, as appropriate. Because the sphingolipids were, for the most part, not normally distributed, nonparametric approaches were used for hypothesis testing. The distributions of sphingolipid levels were compared between SLE cases and controls using Wilcoxon rank sum tests. Median and interquartile ranges (IQR) were calculated for each group, along with racial subgroups (African-Americans and Whites). Bootstrapping was used to compare whether any of the case/control sphingolipid differences in medians were markedly larger for African-Americans or Whites.

Next, we focused on SLE patients alone to examine the extent to which sphingolipid levels differed between patients with and without atherosclerosis and the extent to which sphingolipid levels were correlated with SLEDAI disease activity scores. Wilcoxon rank sum tests were used for between-group comparisons, and Spearman correlations were used when assessing the magnitude of association between sphingolipids and SLEDAI scores. Bootstrapping was used to compare whether any of the observed associations were markedly stronger for African-Americans or Whites.

Since one of the primary goals of this study was one of hypothesis generating, p-values were initially calculated without adjustment for multiple hypothesis testing, and those < 0.05 were considered statistically significant. However, we also reported whether any of the identified significant associations remained significant after adjusting for multiple hypothesis testing using a Benjamini-Hochberg adjusted false discovery rate of 5%. Analyses were conducted using SAS v9.4 (Cary, NC).

## Results

The demographic and clinical characteristics of the study cohort are summarized in Table 1. A similar proportion of SLE patients and controls were African American, and among African-American SLE patients, 63.6% had atherosclerosis. The mean age was about six years older among SLE patients compared with controls (*p* = 0.045). Among the subjects with plasma total cholesterol recorded, the mean was 10% lower among SLE patients compared with controls (181.3 vs. 201.4, *p* = 0.023); concentrations of triglycerides and cholesterol in lipoprotein fractions were similar between SLE patients and controls.

**Table 1:** Demographic and clinical characteristics of study cohort.

	SLE Patients		Control Subjects		*p*
	N = 73		N = 34		
African-Americans [N (%)]		47 (64.4)		22 (64.7)	0.97
Atherosclerosis [N (%)]		38 (52.1)		21 (61.8)	0.35
African-Americans with Atherosclerosis) [N (%)]		30 (63.8)		12 (54.5)	
Age (years) [mean ± SD]		42.11± 14.2	N = 33	36.1 ±13.9	***0*.*045***
SLEDAI total [median (IQR)]		2 (4.0)			
# ACR criteria [median (IQR)]		6 (3.0)			
Lupus nephritis [N (%)]		11 (15.1)			
Patients on Prednisolone [N (%)]		39 (53.4)			
Patients on immune-suppressants [N (%)]					
MMF		25 (34.2)			
CYC		10 (13.7)			
AZA		20 (27.4)			
MTX		10 (13.7)			
Patients on anti-malarials (HCQ) [N (%)]		37 (50.7)			
Patients on biologics (RTX) [N (%)]		5 (6.8)			
Serum creatinine (mg) [mean ± SD]	N = 67	1.2 ± 1.4			
Albumin (g/L) [mean ± SD]	N = 64	3.5 ± 0.6			
Cholesterol (mM) [mean ± SD]	N = 42	181.3 ± 48.4	N = 10	201.4 ± 37.7	***0*.*023***
LDL (mM) [mean ± SD]	N = 42	102.6 ± 40.4	N = 10	128.8 ± 41.0	0.07
HDL (mM) [mean ± SD]	N = 42	54.9 ± 16.7	N = 10	51.4 ± 11.5	0.54
TG (mM) [mean ± SD]	N = 42	124.0 ± 58.6	N = 10	126.8 ± 100.2	0.93

Italicized bold numbers indicate *p* < 0.05

Abbreviations: SLEDAI, Systemic Lupus Erythematosus Disease Activity Index; ACR, American College of Rheumatology score; MMF, mycofenolate; CYC, cyclophosphamide; AZA, azathioprine; MTX, methotrexate; HCQ, hydropxychloroquine; RTX, rituximab; LDL, low-density lipoprotein cholesterol; HDL, high-density lipoprotein cholesterol; TG, triglycerides

### African-American control subjects exhibit different sphingolipid profile compared to White control subjects

Data presented in Table 2 show that African-American control subjects had significantly higher plasma levels of total SM, including C14:0, C16:0, C22:0, C24:1, C24 SM species, compared to White control subjects. Almost all other SM species (except C18:1 SM) exhibited a trend towards higher levels in African-American control subjects compared to White control subjects (Table 2). Conversely, African-American control subjects had significantly lower levels of total Lact-Cer, including the major C14:0, C16:0, C24:1 Lact-Cer species, compared to White control subjects. Levels of the next dominant C22:0 and C24:0 Lact-Cer species exhibited a trend towards lower levels in African-American control subjects as well (Table 2). Many of these differences (i.e. C14:0 SM, C16:0 SM, total SM, C14:0 Lact-Cer, C24:1 Lact-Cer) remained statistically significant after controlling for multiple comparisons.

**Table 2:** Sphingolipid concentration in plasma of healthy White and African-American control subjects.

Sphingolipids [pmol/100 μl]	Control Subjects			
	Whites (W)	African- Americans (AA)	Total	*p* W vs. AA
	N = 12	N = 22	N = 34	
**Sphingomyelin (SM)**				
C14:0 SM	519.9 (408.1, 578.1)	718.5 (608.9, 979.6)	625 (523.2, 825)	***<0*.*001***^**†**^
C16:0 SM	6118.3 (4755.7, 6878.8)	9224.3 (7487.7, 10714)	7956.2 (6463.2, 10060.1)	***<0*.*001***^**†**^
C18:1 SM	2588.3 (1427.3, 2984.2)	2018.3 (1707.5, 2377.8)	2076.7 (1645.4, 2605.2)	0.39
C18:0 SM	757.3 (563.4, 808.8)	774.5 (660.1, 890.3)	768 (631.7, 877.8)	0.31
C20:1 SM	157.5 (137.2, 220.9)	162.8 (142.8, 196.4)	160 (141.2, 200.8)	0.80
C20:0 SM	334.4 (300, 428)	411.1 (356.3, 533.8)	369 (328.5, 515.5)	0.06
C22:1 SM	448.7 (384.8, 568.4)	505.1 (437.7, 638.4)	484.7 (424.3, 618.4)	0.19
C22:0 SM	556.3 (466.2, 607.7)	695.7 (572.7, 799.4)	631.7 (524.3, 757.3)	***0*.*012***
C24:1 SM	1364.2 (1220.3, 1619.2)	1556.1 (1431, 2081.3)	1459 (1333.3, 1797.3)	***0*.*044***
C24:0 SM	420.5 (341.2, 475.7)	551 (410.9, 616.2)	481.4 (375.2, 606.5)	***0*.*037***
C26:1 SM	3.2 (2.8, 3.8)	4 (3.1, 4.6)	3.4 (3, 4.3)	0.1
C26:0 SM	1.3 (1.3, 1.6)	1.5 (1.1, 1.9)	1.4 (1.2, 1.7)	0.26
Total SM	12699.4 (10829.4, 15080.3)	16618.5 (14573.2, 19696.7)	15586 (13123.3, 18852.1)	***0*.*002***^**†**^
**Ceramide (Cer)**				
C14:0 Cer	2.4 (2.2, 2.6)	2.2 (1.6, 2.7)	2.3 (1.7, 2.7)	0.39
C16:0 Cer	9.7 (8.8, 11.3)	10.3 (9.3, 11.4)	10.2 (9.1, 11.4)	0.67
C18:1 Cer	0.3 (0.2, 0.5)	0.6 (0.3, 0.9)	0.5 (0.3, 0.7)	***0*.*007***^**†**^
C18:0 Cer	2.3 (1.6, 3)	2.9 (2.1, 3.9)	2.6 (2.1, 3.6)	0.16
C20:1 Cer	0.5 (0.3, 0.9)	0.7 (0.5, 0.9)	0.6 (0.5, 0.9)	***0*.*033***
C20:0 Cer	7 (5, 8.4)	6.3 (5, 7.1)	6.4 (5, 7.8)	0.69
C22:1 Cer	22.6 (14.5, 25.4)	20.4 (15.2, 25.1)	20.5 (15.2, 25.1)	0.8
C22:0 Cer	73.4 (62.4, 103.8)	81.3 (68.5, 95.1)	80.1 (65.4, 95.2)	0.61
C24:1 Cer	120 (95.5, 143.2)	116.9 (90.3, 126)	117.9 (90.3, 129.5)	0.47
C24:0 Cer	267.4 (200.6, 337.3)	220.5 (198.3, 293.9)	237.4 (198.3, 300.8)	0.35
C26:1 Cer	4.8 (3.9, 6.8)	3.8 (2.7, 4.7)	4.2 (2.8, 5)	0.09
C26:0 Cer	8.9 (6.5, 12.7)	7.1 (6, 9.7)	7.9 (6.1, 10.1)	0.25
Total Cer	527.4 (400.5, 666.4)	477.2 (411.6, 579.4)	488.5 (402.9, 605.1)	0.59
Dihydro-C16:0 Cer	0.3 (0.3, 0.4)	0.4 (0.3, 0.4)	0.4 (0.3, 0.4)	0.12
**Lactosylceramide (Lact-Cer)**				
C14:0 Lact-Cer	37.5 (24.2, 47.6)	18.3 (16.4, 24.9)	22.4 (17.7, 33.5)	***<0*.*001***^**†**^
C16:0 Lact-Cer	830.7 (676.3, 1073.1)	666.2 (526.8, 850)	729.7 (578, 890.5)	***0*.*031***
C18:0 Lact-Cer	15.3 (12.2, 17.8)	17.9 (13.3, 22.2)	17.2 (13.3, 21.7)	0.39
C18:1 Lact-Cer	9.3 (6.8, 12.3)	7.8 (7, 13.5)	8.3 (7, 13)	0.89
C20:0 Lact-Cer	3.6 (2.3, 4.8)	3.3 (2.5, 4.5)	3.5 (2.5, 4.5)	0.97
C20:1 Lact-Cer	0.9 (0.5, 2.2)	1.3 (0.7, 1.7)	1.2 (0.6, 1.7)	0.56
C22:0 Lact-Cer	20.2 (15.7, 23.5)	15 (11.6, 17.8)	16.5 (12.7, 21)	0.07
C22:1 Lact-Cer	1.6 (1.3, 2.2)	1.6 (1.3, 2.2)	1.6 (1.3, 2.2)	0.69
C24:0 Lact-Cer	36.5 (31.3, 42.4)	27.4 (22, 41.2)	31.2 (23.4, 41.8)	0.05
C24:1 Lact-Cer	85.4 (71.6, 118.4)	59.5 (40.2, 88.3)	70.1 (51.3, 100.1)	***0*.*008***^**†**^
C26:0 Lact-Cer	0.4 (0.2, 0.6)	0.4 (0.2, 0.5)	0.4 (0.2, 0.6)	0.64
C26:1 Lact-Cer	0.7 (0.5, 1.1)	0.5 (0.4, 0.9)	0.6 (0.4, 0.9)	0.3
Total Lact-Cer	1035.2 (855.6, 1311.8)	835 (691.6, 1044.2)	915 (749.1, 1082.3)	***0*.*025***
**Hexosylceramide (Hex-Cer)**				
C14:0 Hex-Cer	2.4 (1.5, 3.2)	1.8 (1, 2.1)	1.8 (1.3, 2.4)	0.05
C16:0 Hex-Cer	270 (224.7, 333.1)	223.4 (177.1, 334.8)	247 (194.8, 334.8)	0.39
C18:0 Hex-Cer	6.3 (5.2, 6.9)	5.4 (3.8, 7.6)	5.9 (4.5, 7.4)	0.47
C18:1 Hex-Cer	1.4 (1.1, 1.6)	1.2 (0.9, 1.7)	1.3 (1, 1.6)	0.47
C20:0 Hex-Cer	7.4 (6, 8.7)	4.9 (3.6, 7.8)	5.9 (4.5, 8.1)	0.05
C20:1 Hex-Cer	0.2 (0.1, 0.3)	0.2 (0.1, 0.3)	0.2 (0.1, 0.3)	0.94
C22:0 Hex-Cer	93.8 (80.9, 114.3)	81.8 (60.9, 98.3)	88.8 (65.4, 103.5)	0.08
C22:1 Hex-Cer	1.2 (1, 1.6)	1.3 (1, 1.8)	1.3 (1, 1.7)	0.59
C24:0 Hex-Cer	188.1 (132.2, 259.3)	163.3 (91.6, 229.6)	164.5 (108.8, 235.2)	0.22
C24:1 Hex-Cer	128.4 (87.6, 155.7)	95.2 (72.5, 115.1)	103.5 (77.2, 132.4)	0.05
C26:0 Hex-Cer	2.3 (1.5, 3.3)	1.9 (1.2, 2.3)	2 (1.3, 2.6)	0.16
C26:1 Hex-Cer	0.7 (0.6, 1.2)	0.6 (0.4, 0.9)	0.6 (0.4, 1)	0.3
Total Hex-Cer	697 (543.5, 831.2)	609.5 (502.3, 723)	627.6 (529.8, 782.9)	0.21
**Dihydrosphingosine (dhSph)**	0.6 (0.4, 0.9)	0.9 (0.5, 2.3)	0.8 (0.5, 1.4)	0.07
**Sphingosine**	2.2 (1.4, 2.8)	3.6 (2.1, 17.1)	2.7 (1.9, 5)	**0.025**
**dhSph-1-phosphate (dhSph-1P)**	8.8 (6.9, 13.4)	13.5 (8.8, 22.2)	12.5 (8.2, 17.9)	**0.047**
**Sphingosine 1-phosphate (S1P)**	34.5 (27.4, 46.5)	48.1 (33.8, 77.2)	42.3 (29, 61.3)	0.12
**C16:0 Cer : S1P Ratio**	0.31 (0.24, 0.34)	0.21 (0.13, 0.33)	0.25 (0.15, 0.34)	0.1
**C24:1 Cer : S1P Ratio**	3.22 (2.36, 4.26)	2.4 (1.21, 3.3)	2.75 (1.61, 3.6)	0.08

Italicized bold numbers indicate *p* < 0.05 (not corrected for multiple comparisons)

Data are expressed as median (IQR)]

^**†**^ These p-values remained significant (p<0.05) after adjusting for multiple comparisons as described in the Methods section.

The levels of the least dominant Cer species, C18:1 and C20:1 Cer, were higher in African-American control subjects compared to White control subjects, whereas levels of the remaining Cer species were not different between African-American control subjects and White control subjects (Table 2). Also, levels of sphingosine and dhSph-1P were higher in African-American control subjects. No difference in Hex-Cer levels was identified between African-American control subjects and White control subjects (Table 2). The difference in C18:1 Cer remained statistically significant after controlling for multiple comparisons.

### SLE patients exhibit different sphingolipid profile compared to control subjects

Data presented in Tables 2 and 3 show that irrespective of race, SLE patients had significantly higher plasma levels of total Cer, including C16:0, C18:1, C18:0, C20:1, C:20:0, C24:1, and 26:1 Cer species, compared to control subjects. All other Cer species exhibited a trend towards higher levels in SLE patients compared to control subjects. Furthermore, levels of the sphingoid bases (sphingosine and dhSph) and their phosphates (dhSph-1P and S1P) were significantly higher in SLE patients compared to control subjects. However, the ratios of C16:0 Cer/S1P and C24:1 Cer/S1P were lower in SLE patients. No other major differences in plasma sphingolipid levels were identified between SLE patients and control subjects (Tables 2 and 3). Most of the differences noted in Table 3 were not statistically significant after controlling for multiple comparisons.

**Table 3:** Sphingolipid concentration in plasma of White and African-American SLE patients compared to sphingolipid concentration in plasma of healthy White and African-American control subjects presented in Table 2.

Sphingolipids [pmol/100 μl]	SLE Patients				*p* W SLE vs. W controls	*p* AA SLE vs. AA controls	*p* All SLE vs. All controls	Interaction of SLE with Race *p*<0.05
	Whites (W)	African- Americans (AA)	Total	*p* W vs. AA				
	N = 26	N = 47	N = 73					
**Sphingomyelin (SM)**								
C14:0 SM	519.4 (349.3, 740.2)	623 (487.4, 863.4)	597.9 (462.9, 816.5)	0.06	0.57	0.2	0.59	
C16:0 SM	7155.5 (5709.9, 9488.3)	8378.2 (6160.6, 10875)	7481.1 (6132.4, 10844.7)	0.25	0.1	0.16	0.93	
C18:1 SM	1884.4 (1541.7, 2663.3)	2100.2 (1770.1, 2741.3)	2074.4 (1744.5, 2703.2)	0.39	0.68	0.36	0.7	
C18:0 SM	687.7 (578.3, 948.8)	814.8 (706.6, 910.1)	783.6 (617, 923.1)	0.21	0.88	0.41	0.4	
C20:1 SM	163.4 (139, 206.8)	175 (136.5, 225.6)	172.3 (139, 222.5)	0.29	0.9	0.58	0.76	
C20:0 SM	351.7 (300.3, 467.5)	452.2 (316.4, 524)	411.2 (315.7, 502)	0.08	0.66	0.76	0.95	
C22:1 SM	499.5 (417.4, 618.6)	542.9 (403.6, 668)	536.2 (417.4, 665)	0.21	0.36	0.77	0.47	
C22:0 SM	631.5 (498.1, 890.5)	718.7 (539.8, 867.5)	656 (536.9, 867.5)	0.34	0.17	0.98	0.49	
C24:1 SM	1594.2 (1472.6, 1955.9)	1729.3 (1369.5, 2040.2)	1691.5 (1386.8, 1996.5)	0.56	0.06	0.75	0.18	
C24:0 SM	491.8 (381.5, 635.7)	562.2 (390.4, 700.3)	503.8 (390.4, 689.2)	0.31	0.11	0.98	0.43	
C26:1 SM	3.6 (3.1, 4.6)	4.4 (3.2, 5.1)	4.1 (3.2, 4.9)	0.21	0.12	0.37	0.11	
C26:0 SM	1.5 (1.2, 1.8)	1.6 (1.2, 2)	1.6 (1.2, 1.9)	0.49	0.3	0.69	0.39	
Total SM	14276.1 (11275.3, 17604.8)	16376.1 (13181.1, 19755.9)	15496.6 (12835.9, 18833.9)	0.09	0.27	0.52	0.73	
**Ceramide (Cer)**								
C14:0 Cer	2.7 (2.2, 3.2)	2.3 (1.8, 2.8)	2.6 (2, 3)	***0*.*033***	0.05	0.5	0.12	
C16:0 Cer	11.5 (10, 14.4)	11.9 (10, 17.3)	11.8 (10, 15.4)	0.19	0.06	***0*.*005***	***<0*.*001***^**†**^	
C18:1 Cer	0.5 (0.3, 0.6)	0.7 (0.5, 0.9)	0.6 (0.4, 0.9)	***0*.*006***	***0*.*016***	0.27	***0*.*03***	
C18:0 Cer	3 (2.2, 3.9)	3.4 (2.8, 4.4)	3.4 (2.5, 4.2)	0.14	0.06	0.07	***0*.*009***	
C20:1 Cer	0.8 (0.6, 1)	0.9 (0.6, 1.1)	0.9 (0.6, 1.1)	0.18	***0*.*028***	***0*.*046***	***0*.*006***	
C20:0 Cer	8.1 (6.7, 9.8)	7.5 (6, 8.8)	7.8 (6.3, 8.8)	0.21	0.09	0.06	***0*.*01***	
C22:1 Cer	23.5 (19.8, 28.9)	22.5 (16.7, 30.8)	22.9 (18.5, 28.9)	0.34	0.12	0.2	0.06	
C22:0 Cer	94.2 (77.5, 111.7)	97.1 (65.2, 118.4)	95.3 (69.1, 117.5)	0.64	0.1	0.25	0.05	
C24:1 Cer	126.7 (108.2, 167)	125.2 (100.7, 176.6)	126.3 (103.1, 170.1)	0.71	0.19	0.07	***0*.*022***	
C24:0 Cer	299.1 (228.9, 368.4)	265.5 (210.7, 373.1)	282.8 (215.8, 368.4)	0.52	0.41	0.18	0.11	
C26:1 Cer	5.6 (4.2, 6.5)	4.8 (4.2, 7)	5.2 (4.2, 6.6)	0.64	0.62	***0*.*008***	***0*.*013***	
C26:0 Cer	8.7 (7.6, 14.4)	8.7 (5.9, 11.3)	8.7 (6.7, 12.8)	0.41	0.8	0.35	0.37	
Total Cer	606.3 (472.9, 720.5)	545.6 (441.7, 752.5)	553.5 (472.9, 720.5)	0.62	0.21	0.1	***0*.*036***	
Dihydro-C16:0 Cer	0.3 (0.3, 0.4)	0.4 (0.3, 0.5)	0.4 (0.3, 0.4)	0.06	0.29	0.55	0.45	
**Lactosylceramide (Lact-Cer)**								
C14:0 Lact-Cer	27.9 (23.1, 34.7)	17.9 (14, 25.6)	22.3 (14.8, 30.2)	***<0*.*001***^**†**^	0.22	0.46	0.37	
C16:0 Lact-Cer	838.6 (646.9, 987)	639.7 (486.9, 835.8)	713.2 (540.2, 898.1)	***0*.*006***	0.68	0.64	0.59	
C18:0 Lact-Cer	12.6 (6.6, 20.4)	21.3 (16.3, 28.7)	18.6 (12.3, 26.2)	***<0*.*001***^**†**^	0.27	0.06	0.36	
C18:1 Lact-Cer	10 (7.5, 14.3)	9.9 (8, 14.5)	9.9 (7.9, 14.3)	0.95	0.45	0.27	0.2	
C20:0 Lact-Cer	2.8 (1.8, 4.2)	3.5 (2.6, 4.8)	3.2 (2.3, 4.4)	***0*.*031***	0.36	0.6	0.87	
C20:1 Lact-Cer	0.9 (0.4, 1.4)	1.2 (0.8, 2.1)	1.1 (0.7, 1.8)	***0*.*041***	0.66	0.46	0.84	
C22:0 Lact-Cer	16.8 (12.3, 19.1)	14.3 (11.7, 20.3)	15.2 (11.9, 19.3)	0.64	0.19	0.66	0.67	
C22:1 Lact-Cer	1.8 (1.4, 2.3)	1.8 (1.3, 2.5)	1.8 (1.3, 2.4)	0.7	0.83	0.25	0.28	
C24:0 Lact-Cer	30.9 (24.2, 39)	31 (24.8, 38.3)	31 (24.8, 38.5)	0.67	0.16	0.52	0.63	
C24:1 Lact-Cer	88.3 (68.2, 102.1)	72.1 (56.6, 85.1)	74.2 (58.6, 91.7)	***0*.*021***	0.64	0.25	0.7	
C26:0 Lact-Cer	0.4 (0.1, 0.6)	0.5 (0.3, 0.7)	0.4 (0.2, 0.7)	0.29	0.92	0.14	0.38	
C26:1 Lact-Cer	0.6 (0.4, 0.8)	0.5 (0.3, 0.8)	0.6 (0.4, 0.8)	0.32	0.4	0.58	0.37	
Total Lact-Cer	1034.6 (837.8, 1205.7)	808.3 (675.4, 1004.6)	914.1 (709.4, 1105.4)	***0*.*012***	0.57	0.78	0.64	
**Hexosylceramide (Hex-Cer)**								
C14:0 Hex-Cer	2.1 (1.5, 3.3)	1.6 (1.1, 2.2)	1.8 (1.4, 2.6)	***0*.*018***	0.97	0.79	0.68	
C16:0 Hex-Cer	262.4 (211.7, 310.5)	260.5 (200.1, 407.3)	260.5 (211.7, 368.1)	0.61	0.83	0.24	0.44	
C18:0 Hex-Cer	7.8 (4.6, 10.7)	6.5 (4.7, 9.2)	6.8 (4.7, 9.3)	0.42	0.23	0.16	0.07	
C18:1 Hex-Cer	1.2 (0.9, 1.7)	1.5 (1, 2.2)	1.4 (0.9, 2)	0.13	0.33	0.2	0.51	
C20:0 Hex-Cer	7.7 (4.5, 9.5)	6.3 (5.3, 9.3)	6.4 (5.1, 9.3)	0.94	0.85	***0*.*03***	0.17	
C20:1 Hex-Cer	0.1 (0.1, 0.2)	0.4 (0.2, 0.5)	0.3 (0.1, 0.4)	***<0*.*001***^**†**^	0.35	***0*.*004***	0.07	**YES**
C22:0 Hex-Cer	95.9 (62.8, 115.1)	87.5 (71.7, 118.5)	90.1 (70.4, 115.1)	0.87	0.71	0.1	0.28	
C22:1 Hex-Cer	1.6 (1.1, 1.9)	2.1 (1.4, 2.5)	1.7 (1.4, 2.2)	***0*.*013***	0.23	***0*.*004***	***0*.*003***	
C24:0 Hex-Cer	170.7 (100.8, 247.7)	181.6 (128.5, 253)	176.2 (124.5, 248.5)	0.26	0.51	0.1	0.37	
C24:1 Hex-Cer	121.4 (76.7, 155.6)	112.4 (91.6, 154.3)	115.5 (86.9, 154.3)	0.84	0.64	***0*.*02***	0.11	
C26:0 Hex-Cer	1.9 (1.1, 3.1)	2.6 (1.9, 3.5)	2.3 (1.6, 3.1)	0.05	0.38	***0*.*007***	0.11	
C26:1 Hex-Cer	0.8 (0.5, 1.1)	0.8 (0.5, 1.2)	0.8 (0.5, 1.2)	0.52	1	***0*.*042***	0.1	
Total Hex-Cer	710.8 (504, 867.4)	673.6 (596.9, 874.9)	675.5 (560.4, 869.2)	0.58	0.8	0.08	0.24	
**Dihydrosphingosine (dhSph)**	0.8 (0.6, 1.2)	1.3 (0.7, 2.4)	1 (0.7, 1.8)	***0*.*01***	***0*.*006***	0.17	***0*.*014***	
**Sphingosine**	4 (2.2, 7.8)	6.4 (2.7, 15.8)	5.1 (2.5, 11.9)	***0*.*04***	***<0*.*001***^**†**^	***0*.*013***	***<0*.*001***^**†**^	
**dhSph 1-phosphate**	20.8 (17.3, 24.3)	17.5 (13.6, 29.3)	19.1 (15.7, 24.8)	0.24	***<0*.*001***^**†**^	***0*.*007***	***<0*.*001***^**†**^	**YES**
**Sphingosine 1-phosphate (S1P)**	72.1 (62.2, 87.5)	68.9 (53.7, 85.8)	68.9 (55.7, 87)	0.34	***<0*.*001***^**†**^	0.37	***0*.*005***	
**C16:0 Cer : S1P Ratio**	0.14 (0.13, 0.18)	0.19 (0.14, 0.25)	0.17 (0.13, 0.23)	0.06	***<0*.*001***^**†**^	0.35	***0*.*006***	**YES**
**C24:1 Cer : S1P Ratio**	1.78 (1.46, 2.11)	1.83 (1.5, 2.56)	1.82 (1.5, 2.45)	0.4	***<0*.*001***^**†**^	0.35	***0*.*006***	

Italicized bold numbers indicate *p* < 0.05 (not corrected for multiple comparisons)

Data are expressed as median (IQR)]

^**†**^ These p-values remained significant (p<0.05) after adjusting for multiple comparisons as described in the Methods section.

### African-American SLE patients and White SLE patients exhibit different sphingolipid profile compared to their respective control subjects

Data presented in Tables 2 and 3 show that African-American SLE patients had significantly higher plasma levels of C16:0, C20:1, and C26:1 Cer species, compared to African-American control subjects. All other Cer species and total Cer exhibited a trend towards higher levels in African-American SLE patients compared to African-American control subjects. Interestingly, African-American SLE patients had significantly higher plasma levels of C20:0, C20:1, C22:1, C24:1, C24:1, and C26:1 Hex-Cer species, compared to African-American control subjects. All other Hex-Cer species and total Hex-Cer exhibited a trend towards higher levels in African-American SLE patients compared to African-American control subjects. Among the sphingoid bases and their phosphates, levels of sphingosine and dhSph-1P were significantly higher in African-American SLE patients compared to their respective control subjects. No other major differences in plasma sphingolipid levels were identified between African-American SLE patients and African-American control subjects (Tables 2 and 3).

White SLE patients, on the other hand, had significantly higher plasma levels of C18:1 and C20:1 Cer species, compared to White control subjects. Similar to African-Americans, White SLE patients exhibited a trend towards higher levels of total Cer and almost all other Cer species (except C26 Cer) compared to White control subjects (Tables 2 and 3). Similar to the whole cohort data, levels of the sphingoid bases (sphingosine and dhSph) and their phosphates (dhSph-1P and S1P) were significantly higher in White SLE patients compared to White control subjects, whereas the ratios of C16:0 Cer/S1P and C24:1 Cer/S1P were lower in White SLE patients compared to White control subjects. No other major differences in plasma sphingolipid levels were identified between White SLE patients and White control subjects (Tables 2 and 3).

### White SLE patients with atherosclerosis exhibit lower sphingoid bases levels compared to White SLE patients without atherosclerosis

Data presented in Table 4 show that the White SLE patients with atherosclerosis exhibited no significant difference in any of the measured circulating sphingolipids compared to the White SLE patients without atherosclerosis except for the sphingoid bases. White SLE patients with atherosclerosis had significantly lower sphingosine and dhSph than the White SLE patients without atherosclerosis (Table 4), although these differences did not remain significant after controlling for multiple comparisons.

**Table 4:** Sphingolipid concentration in plasma of White SLE patients with atherosclerosis compared to sphingolipid concentration in plasma of White SLE patients without atherosclerosis.

Sphingolipids	White (W) SLE Subjects			
	No Atherosclerosis (No Athero)	With Atherosclerosis (Athero)	Total	*p* Athero vs. No Athero
	N = 18	N = 8	N = 26	
**Sphingomyelin (SM)**				
C14:0 SM	519.4 (318.4, 678.2)	532.2 (404.4, 771.7)	519.4 (349.3, 740.2)	0.5
C16:0 SM	6761.7 (5030, 8486.6)	7653.2 (6734.5, 10303.8)	7155.5 (5709.9, 9488.3)	0.22
C18:1 SM	1864.6 (1541.7, 2663.3)	2165.4 (1450, 2899.5)	1884.4 (1541.7, 2663.3)	0.78
C18:0 SM	676.2 (578.3, 948.8)	810.7 (566.5, 992.6)	687.7 (578.3, 948.8)	0.62
C20:1 SM	151.2 (139, 223.6)	186.3 (133.2, 206.3)	163.4 (139, 206.8)	0.78
C20:0 SM	338.7 (300.3, 467.5)	379.6 (324.6, 447.6)	351.7 (300.3, 467.5)	0.82
C22:1 SM	478.6 (380.2, 646.7)	543.8 (481.8, 588.9)	499.5 (417.4, 618.6)	0.54
C22:0 SM	603.1 (437.8, 913.6)	652.3 (599.3, 804.7)	631.5 (498.1, 890.5)	0.62
C24:1 SM	1536.1 (1313.1, 1931.3)	1831.7 (1521.8, 2001)	1594.2 (1472.6, 1955.9)	0.4
C24:0 SM	466.3 (362.6, 697.1)	499.4 (484.7, 600.5)	491.8 (381.5, 635.7)	0.4
C26:1 SM	3.6 (3.1, 4.5)	4.2 (3.3, 5.1)	3.6 (3.1, 4.6)	0.34
C26:0 SM	1.4 (1, 1.9)	1.6 (1.3, 1.8)	1.5 (1.2, 1.8)	0.58
Total SM	13550.8 (10744.7, 17604.8)	15331.7 (13787.7, 18593.9)	14276.1 (11275.3, 17604.8)	0.24
**Ceramide (Cer)**				
C14:0 Cer	2.7 (2.2, 3.3)	2.7 (2.3, 2.9)	2.7 (2.2, 3.2)	0.74
C16:0 Cer	11.5 (10, 13.9)	12.2 (9.8, 15.6)	11.5 (10, 14.4)	0.62
C18:1 Cer	0.5 (0.3, 0.6)	0.6 (0.4, 0.7)	0.5 (0.3, 0.6)	0.47
C18:0 Cer	3.1 (2.1, 3.9)	2.9 (2.3, 3.8)	3 (2.2, 3.9)	0.87
C20:1 Cer	0.8 (0.6, 1)	0.7 (0.6, 1)	0.8 (0.6, 1)	0.74
C20:0 Cer	8.3 (7.2, 9.8)	7.2 (5.9, 9.6)	8.1 (6.7, 9.8)	0.29
C22:1 Cer	24.7 (18.6, 28.9)	23.3 (20.7, 26.9)	23.5 (19.8, 28.9)	0.87
C22:0 Cer	92.8 (77.5, 111.7)	95.7 (81.5, 109.3)	94.2 (77.5, 111.7)	0.82
C24:1 Cer	126.5 (113.1, 156.4)	128.1 (107.3, 185.4)	126.7 (108.2, 167)	0.78
C24:0 Cer	296.1 (229.1, 368.4)	317.2 (204.7, 396.4)	299.1 (228.9, 368.4)	0.74
C26:1 Cer	5.6 (3.7, 6.1)	5.9 (4.9, 7.1)	5.6 (4.2, 6.5)	0.4
C26:0 Cer	9.1 (7.6, 14.4)	8.2 (7.8, 13.6)	8.7 (7.6, 14.4)	0.91
Total Cer	606.3 (480.9, 648.3)	616 (445.2, 751.3)	606.3 (472.9, 720.5)	0.82
Dihydro-C16:0 Cer	0.3 (0.3, 0.4)	0.3 (0.3, 0.4)	0.3 (0.3, 0.4)	0.11
**Lactosylceramide (Lact-Cer)**				
C14:0 Lact-Cer	30.7 (23.1, 34.9)	25.4 (22, 28.2)	27.9 (23.1, 34.7)	0.24
C16:0 Lact-Cer	891.5 (646.9, 1056.6)	774.1 (646.8, 929.3)	838.6 (646.9, 987)	0.62
C18:0 Lact-Cer	13.1 (9.5, 22.8)	9.2 (4.3, 17.5)	12.6 (6.6, 20.4)	0.15
C18:1 Lact-Cer	13.3 (7.4, 14.4)	9.6 (8.2, 10)	10 (7.5, 14.3)	0.47
C20:0 Lact-Cer	3 (2.3, 4.2)	2.1 (1.2, 2.9)	2.8 (1.8, 4.2)	0.08
C20:1 Lact-Cer	0.9 (0.5, 1.4)	0.7 (0.3, 1.4)	0.9 (0.4, 1.4)	0.58
C22:0 Lact-Cer	16.2 (12.9, 20)	16.9 (11.2, 17.9)	16.8 (12.3, 19.1)	0.66
C22:1 Lact-Cer	1.8 (1.2, 2.3)	1.7 (1.4, 2.2)	1.8 (1.4, 2.3)	0.82
C24:0 Lact-Cer	30.9 (26.9, 42.2)	30.2 (14.1, 38.6)	30.9 (24.2, 39)	0.27
C24:1 Lact-Cer	92.5 (68.2, 108.8)	81.7 (64.7, 93.7)	88.3 (68.2, 102.1)	0.4
C26:0 Lact-Cer	0.3 (0.1, 0.6)	0.5 (0.4, 0.7)	0.4 (0.1, 0.6)	0.27
C26:1 Lact-Cer	0.6 (0.5, 0.8)	0.5 (0.3, 1.1)	0.6 (0.4, 0.8)	0.54
Total Lact-Cer	1098.1 (837.8, 1261.4)	947.8 (828.7, 1099.7)	1034.6 (837.8, 1205.7)	0.5
**Hexosylceramide (Hex-Cer)**				
C14:0 Hex-Cer	1.9 (1.5, 3.2)	2.7 (1.7, 3.5)	2.1 (1.5, 3.3)	0.44
C16:0 Hex-Cer	262.4 (216.8, 309.4)	272.9 (178, 366.8)	262.4 (211.7, 310.5)	0.91
C18:0 Hex-Cer	7.4 (4.6, 8.4)	9.4 (5.3, 11.3)	7.8 (4.6, 10.7)	0.4
C18:1 Hex-Cer	1.2 (0.9, 1.6)	1.2 (0.8, 1.7)	1.2 (0.9, 1.7)	0.87
C20:0 Hex-Cer	6.8 (4.5, 8.5)	8.3 (3.2, 10.1)	7.7 (4.5, 9.5)	0.87
C20:1 Hex-Cer	0.2 (0.1, 0.2)	0.1 (0, 0.3)	0.1 (0.1, 0.2)	0.47
C22:0 Hex-Cer	84.4 (61.5, 115.1)	98.3 (95.9, 110.3)	95.9 (62.8, 115.1)	0.4
C22:1 Hex-Cer	1.6 (1.1, 1.9)	1.6 (1.2, 1.9)	1.6 (1.1, 1.9)	0.87
C24:0 Hex-Cer	168.2 (101.2, 234.5)	185.8 (65.4, 253.3)	170.7 (100.8, 247.7)	0.62
C24:1 Hex-Cer	121.4 (72, 155.6)	117.6 (96.8, 150.7)	121.4 (76.7, 155.6)	0.82
C26:0 Hex-Cer	1.9 (1.4, 3.1)	2.1 (0.5, 3.1)	1.9 (1.1, 3.1)	0.7
C26:1 Hex-Cer	0.7 (0.4, 1.1)	1 (0.7, 1.2)	0.8 (0.5, 1.1)	0.47
Total Hex-Cer	674.4 (504, 867.4)	732.1 (507.4, 874.1)	710.8 (504, 867.4)	0.82
**Dihydrosphingosine (dhSph)**	1 (0.7, 1.3)	0.6 (0.5, 0.7)	0.8 (0.6, 1.2)	***0*.*011***
**Sphingosine**	4.7 (3.1, 8.8)	2.2 (1.7, 3.2)	4 (2.2, 7.8)	***0*.*008***
**dhSph-1-phosphate (dhSph-1P)**	21.9 (18.2, 24.3)	19 (16.5, 20.5)	20.8 (17.3, 24.3)	0.09
**Sphingosine 1-phosphate (S1P)**	79.9 (65.4, 98.6)	61.7 (58.2, 76.7)	72.1 (62.2, 87.5)	0.07
**C16:0 Cer : S1P Ratio**	0.14 (0.13, 0.17)	0.17 (0.15, 0.23)	0.14 (0.13, 0.18)	0.09
**C24:1 Cer : S1P Ratio**	1.68 (1.46, 1.97)	1.97 (1.63, 2.56)	1.78 (1.46, 2.11)	0.18

Italicized bold numbers indicate *p* < 0.05 (not corrected for multiple comparisons)

Data are expressed as median (IQR)]

### African-American SLE patients with atherosclerosis exhibit different sphingolipid profile compared to African-American SLE patients without atherosclerosis

Data presented in Table 5 show that the African-American SLE patients with atherosclerosis had significantly higher plasma levels of total SM, including C16:0, C18:0, C20:1, C20:0, C22:1, C22:0, C24:1, and C26:1 SM species, compared to African-American SLE patients without atherosclerosis. The remaining four measured SM species exhibited a trend towards higher levels in African-American SLE patients with atherosclerosis as well (Table 5). Unlike Whites (Table 4), African-American SLE patients with atherosclerosis had significantly higher levels of sphingosine and dhSph than the African-American SLE patients without atherosclerosis (Table 5). Whereas levels of C24:1 Lact-Cer, were significantly lower in African-American SLE patients with atherosclerosis compared to those without, levels of C18:1 Lact-Cer were significantly higher in African-American SLE patients with atherosclerosis (Table 5). No differences in Cer levels were identified between African-American SLE patients with atherosclerosis and those without (Table 5). Although several findings in Table 5 were statistically significant in the unadjusted comparisons, most did not remain statistically significant after controlling for multiple comparisons.

**Table 5:** Sphingolipid concentration in plasma of African-American SLE patients with atherosclerosis compared to sphingolipid concentration in plasma of African-American SLE patients without atherosclerosis and to sphingolipid concentration in White SLE patients with atherosclerosis presented in Table 4.

Sphingolipids	African-American (AA) SLE Patients				*p* W SLE Athero vs. AA SLE Athero	Interaction of SLE Athero with Race *p*<0.05
	No Atherosclerosis (No Athero)	With Atherosclerosis (Athero)	Total	*p* Athero vs. No Athero		
	N = 17	N = 30	N = 47			
**Sphingomyelin (SM)**						
C14:0 SM	530 (493.3, 621.6)	716.1 (485.2, 985.9)	623 (487.4, 863.4)	0.06	0.19	
C16:0 SM	6836.3 (6132.4, 8091.9)	9919.5 (6272.6, 11337.7)	8378.2 (6160.6, 10875)	***0*.*032***	0.62	
C18:1 SM	1948.2 (1695.8, 2267.7)	2339.1 (1812.8, 2837.5)	2100.2 (1770.1, 2741.3)	0.11	0.54	
C18:0 SM	756.2 (605.9, 835.8)	887.4 (727.4, 937.2)	814.8 (706.6, 910.1)	***0*.*027***	0.45	
C20:1 SM	147.8 (124.4, 178.8)	206.7 (154.6, 234)	175 (136.5, 225.6)	***0*.*007***	0.16	
C20:0 SM	348.4 (312.5, 464.5)	478 (346.8, 559.6)	452.2 (316.4, 524)	***0*.*044***	0.07	
C22:1 SM	470.9 (388.6, 578.1)	582.1 (485, 716.8)	542.9 (403.6, 668)	***0*.*028***	0.21	
C22:0 SM	557.3 (524.2, 824.8)	779.3 (613.8, 907.8)	718.7 (539.8, 867.5)	***0*.*049***	0.33	
C24:1 SM	1386.8 (1142.9, 1941)	1802.6 (1597.9, 2313.1)	1729.3 (1369.5, 2040.2)	***0*.*012***	0.67	
C24:0 SM	432.6 (384.6, 618.1)	600.6 (432.9, 701.4)	562.2 (390.4, 700.3)	0.07	0.25	
C26:1 SM	3.3 (2.7, 4.7)	4.5 (4, 5.7)	4.4 (3.2, 5.1)	***0*.*01***	0.33	
C26:0 SM	1.4 (1.1, 1.9)	1.6 (1.4, 2)	1.6 (1.2, 2)	0.12	0.67	
Total SM	14271 (12182.8, 15498.3)	17194.9 (15570.9, 21360)	16376.1 (13181.1, 19755.9)	***0*.*005***	0.21	
**Ceramide (Cer)**						
C14:0 Cer	2.3 (1.8, 2.8)	2.4 (1.9, 2.8)	2.3 (1.8, 2.8)	0.79	0.27	
C16:0 Cer	12.6 (10.1, 16.7)	11.8 (10, 17.3)	11.9 (10, 17.3)	0.95	0.67	
C18:1 Cer	0.6 (0.4, 0.8)	0.7 (0.5, 0.9)	0.7 (0.5, 0.9)	0.44	0.12	
C18:0 Cer	3.4 (2.8, 4.4)	3.4 (2.8, 4.3)	3.4 (2.8, 4.4)	0.95	0.22	
C20:1 Cer	0.8 (0.6, 1.1)	1 (0.7, 1.1)	0.9 (0.6, 1.1)	0.3	0.24	
C20:0 Cer	6.8 (5.5, 8.9)	7.6 (6.4, 8.6)	7.5 (6, 8.8)	0.47	0.83	
C22:1 Cer	22.9 (18.8, 27.7)	21.2 (16.5, 30.8)	22.5 (16.7, 30.8)	0.86	0.59	
C22:0 Cer	98.9 (61.6, 109.8)	89.3 (67, 121.5)	97.1 (65.2, 118.4)	0.93	0.57	
C24:1 Cer	133.9 (100.7, 169.8)	124.9 (103.1, 183.2)	125.2 (100.7, 176.6)	0.81	0.57	
C24:0 Cer	277.4 (210.7, 415.3)	261.8 (215.8, 345.3)	265.5 (210.7, 373.1)	0.66	0.54	
C26:1 Cer	5 (4.1, 7.4)	4.7 (4.2, 6.9)	4.8 (4.2, 7)	0.98	0.37	
C26:0 Cer	10.3 (6.3, 14)	8.4 (5.3, 9.5)	8.7 (5.9, 11.3)	0.16	0.45	
Total Cer	582.4 (418.7, 752.5)	535.1 (473.1, 691.5)	545.6 (441.7, 752.5)	0.66	0.62	
Dihydro-C16:0 Cer	0.4 (0.3, 0.4)	0.4 (0.3, 0.5)	0.4 (0.3, 0.5)	0.77	0.06	
**Lactosylceramide (Lact-Cer)**						
C14:0 Lact-Cer	21 (16.7, 29.5)	15.7 (13.1, 25.4)	17.9 (14, 25.6)	0.22	0.1	
C16:0 Lact-Cer	713.2 (635.1, 863.1)	548.5 (431.5, 821.3)	639.7 (486.9, 835.8)	0.06	0.05	
C18:0 Lact-Cer	18.9 (17, 26.7)	25 (12.9, 29.4)	21.3 (16.3, 28.7)	0.77	***0*.*005***	
C18:1 Lact-Cer	8.2 (7.9, 9.9)	12 (9.2, 15.4)	9.9 (8, 14.5)	***0*.*015***	0.16	**YES**
C20:0 Lact-Cer	3.5 (2.9, 4.7)	3.7 (2.3, 4.8)	3.5 (2.6, 4.8)	0.76	***0*.*02***	
C20:1 Lact-Cer	1 (0.9, 2.1)	1.4 (0.7, 2.1)	1.2 (0.8, 2.1)	0.76	0.11	
C22:0 Lact-Cer	16.8 (13.5, 19.3)	14 (11.6, 20.5)	14.3 (11.7, 20.3)	0.49	0.8	
C22:1 Lact-Cer	1.5 (1.3, 2.1)	1.9 (1.5, 2.5)	1.8 (1.3, 2.5)	0.45	0.45	
C24:0 Lact-Cer	33.3 (26.1, 39.6)	29.7 (24.1, 36.2)	31 (24.8, 38.3)	0.1	0.83	
C24:1 Lact-Cer	79.2 (72.4, 102.7)	63 (48.7, 72.8)	72.1 (56.6, 85.1)	***0*.*008***	0.09	
C26:0 Lact-Cer	0.7 (0.4, 0.8)	0.4 (0.2, 0.5)	0.5 (0.3, 0.7)	***0*.*014***	0.25	**YES**
C26:1 Lact-Cer	0.6 (0.6, 0.8)	0.4 (0.3, 0.7)	0.5 (0.3, 0.8)	***0*.*024***	0.67	
Total Lact-Cer	930.7 (764.5, 1089.3)	733.1 (570.7, 949.9)	808.3 (675.4, 1004.6)	0.05	0.09	
**Hexosylceramide (Hex-Cer)**						
C14:0 Hex-Cer	1.4 (1.2, 2.2)	1.7 (1, 2.2)	1.6 (1.1, 2.2)	0.66	0.06	
C16:0 Hex-Cer	301.7 (246.8, 379.6)	247.8 (179.4, 407.3)	260.5 (200.1, 407.3)	0.14	0.91	
C18:0 Hex-Cer	5.4 (4.7, 7.4)	6.9 (5.5, 10.5)	6.5 (4.7, 9.2)	0.22	0.47	
C18:1 Hex-Cer	1.3 (0.9, 1.5)	1.7 (1.2, 2.3)	1.5 (1, 2.2)	0.19	0.19	
C20:0 Hex-Cer	5.9 (5.3, 10.2)	6.5 (5.3, 8.5)	6.3 (5.3, 9.3)	0.77	0.83	
C20:1 Hex-Cer	0.2 (0.1, 0.3)	0.5 (0.3, 0.6)	0.4 (0.2, 0.5)	***<0*.*001***^**†**^	***0*.*002***	**YES**
C22:0 Hex-Cer	84 (73.1, 126.2)	88.8 (71.7, 109.3)	87.5 (71.7, 118.5)	0.96	0.54	
C22:1 Hex-Cer	1.6 (1.4, 2.1)	2.1 (1.6, 2.8)	2.1 (1.4, 2.5)	***0*.*034***	***0*.*041***	
C24:0 Hex-Cer	196.4 (124.5, 262)	175.9 (139.6, 248.5)	181.6 (128.5, 253)	0.64	0.57	
C24:1 Hex-Cer	110.7 (96.1, 130.1)	116.1 (86.9, 154.3)	112.4 (91.6, 154.3)	0.91	0.89	
C26:0 Hex-Cer	2.2 (1.9, 3)	2.6 (1.9, 3.5)	2.6 (1.9, 3.5)	0.52	0.19	
C26:1 Hex-Cer	0.8 (0.6, 1.2)	0.8 (0.5, 1.2)	0.8 (0.5, 1.2)	0.86	0.75	
Total Hex-Cer	732.8 (608.6, 887.3)	670.8 (560.4, 869.2)	673.6 (596.9, 874.9)	0.58	0.89	
**Dihydrosphingosine (dhSph)**	1 (0.6, 1.4)	1.8 (0.8, 5.2)	1.3 (0.7, 2.4)	***0*.*014***	***0*.*002***	**YES**^**†**^
**Sphingosine**	4.4 (2.7, 6)	9.2 (3.4, 29.6)	6.4 (2.7, 15.8)	***0*.*037***	***0*.*004***	**YES**^**†**^
**dhSph 1-phosphate**	17.9 (15.4, 27.8)	17.1 (11.9, 30.3)	17.5 (13.6, 29.3)	0.39	0.8	
**Sphingosine 1-phosphate (S1P)**	67.6 (55.7, 85.8)	69.8 (51.5, 83.6)	68.9 (53.7, 85.8)	0.61	0.83	
**C16:0 Cer : S1P Ratio**	0.17 (0.14, 0.25)	0.2 (0.14, 0.25)	0.19 (0.14, 0.25)	0.72	0.72	
**C24:1 Cer : S1P Ratio**	1.80 (1.50, 2.56)	2.02 (1.52, 2.56)	1.83 (1.5, 2.56)	0.66	0.75	

Italicized bold numbers indicate *p* < 0.05 (not corrected for multiple comparisons)

Data are expressed as median (IQR)]

^**†**^ These p-values remained significant (p<0.05) after adjusting for multiple comparisons as described in the Methods section.

### African-American SLE patients with atherosclerosis exhibit higher levels of the sphingoid bases and select Lact-Cer and Hex-Cer species compared to White SLE patients with atherosclerosis

Data presented in Table 5 show that the African-American SLE patients with atherosclerosis had significantly higher plasma levels of the sphingoid bases (sphingosine and dhSph), and also higher levels of two Lact-Cer species, C18:0 and C20:0, and two Hex-Cer species, C20:1 and C22:1. These differences existed also among the significant differences in sphingolipid levels between the whole White SLE patients and African-American SLE patients as shown in Table 3. No other differences in plasma sphingolipid levels were identified between African-American SLE patients with atherosclerosis and White SLE patients with atherosclerosis (Table 5).

### The effect of interaction of SLE with race on the median plasma sphingolipid levels

Data presented in Table 3 show that the effect of SLE on levels of C20:1 Hex-Cer, dhSph-1P, and the C16:0 Cer/S1P Ratio could be dependent on race, African-Americans *versus* Whites. Furthermore, data presented in Table 5 show that the effect of SLE associated with atherosclerosis on levels of C18:1 and C26:0 Lact-Cer, C20:1 Hex-Cer, and the sphingoid bases (sphingosine and dhSph) could also be dependent on race.

### Correlation of plasma sphingolipid levels with SLEDAI in White and African-American SLE patients

Data presented in Table 6 show that plasma levels of sphingosine, C16:0 Cer/S1P ratio and C24:1 Cer/S1P ratio significantly correlated with SLEDAI in African-American SLE patients, although these findings were not significant after adjusting for multiple comparisons. There was no correlation between SLEDAI and sphingolipid levels in White SLE patients or in the whole SLE cohort, irrespective of race (Table 6). The differences in correlation of sphingolipid species levels with SLEDAI between White and African-American SLE patients were found to be not statistically significant.

**Table 6:** Correlation of sphingolipid concentration with Systemic Lupus Erythematosus Disease Activity Index (SLEDAI) score for White and African-American SLE patients.

Sphingolipids	Total SLE Patients		White (W)		African-American (AA)	
	N = 73		N = 26		N = 47	
	Spearman Correlation	*p*	Spearman Correlation	*p*	Spearman Correlation	*p*
**Sphingomyelin (SM)**						
C14:0 SM	-0.06	0.602	-0.07	0.726	-0.11	0.479
C16:0 SM	-0.03	0.818	-0.08	0.681	-0.04	0.817
C18:1 SM	0.07	0.539	0	0.982	0.09	0.542
C18:0 SM	0.04	0.747	-0.19	0.362	0.12	0.444
C20:1 SM	0.06	0.646	-0.09	0.649	0.09	0.549
C20:0 SM	0.01	0.901	-0.26	0.205	0.11	0.454
C22:1 SM	0.05	0.694	-0.11	0.581	0.1	0.523
C22:0 SM	0	0.987	-0.17	0.415	0.07	0.643
C24:1 SM	0.05	0.666	-0.11	0.585	0.1	0.517
C24:0 SM	0	0.979	-0.17	0.403	0.05	0.736
C26:1 SM	-0.01	0.954	-0.16	0.44	0.02	0.88
C26:0 SM	0.04	0.762	-0.14	0.508	0.11	0.475
Total SM	0	0.99	-0.12	0.558	0.01	0.971
**Ceramide (Cer)**						
C14:0 Cer	0.08	0.498	-0.03	0.886	0.15	0.309
C16:0 Cer	0.18	0.127	-0.06	0.775	0.28	0.057
C18:1 Cer	0.08	0.527	0.05	0.814	0.08	0.584
C18:0 Cer	0.15	0.224	0.05	0.808	0.19	0.197
C20:1 Cer	0.16	0.176	0.14	0.502	0.2	0.178
C20:0 Cer	0.13	0.279	0.19	0.342	0.1	0.498
C22:1 Cer	0.11	0.368	0.07	0.717	0.16	0.286
C22:0 Cer	0.06	0.602	-0.02	0.907	0.11	0.452
C24:1 Cer	0.19	0.12	0.07	0.727	0.25	0.095
C24:0 Cer	0.08	0.523	0.07	0.723	0.1	0.489
C26:1 Cer	0.12	0.332	0.08	0.681	0.15	0.335
C26:0 Cer	0.09	0.452	0.13	0.541	0.1	0.513
Total Cer	0.12	0.332	0.05	0.79	0.15	0.304
Dihydro-C16:0 Cer	0.2	0.091	0.08	0.713	0.27	0.073
**Lactosylceramide (Lact-Cer)**						
C14:0 Lact-Cer	-0.1	0.405	-0.23	0.26	0.06	0.709
C16:0 Lact-Cer	-0.06	0.612	-0.26	0.209	0.1	0.53
C18:0 Lact-Cer	0.12	0.319	-0.05	0.802	0.18	0.233
C18:1 Lact-Cer	-0.09	0.433	-0.03	0.893	-0.09	0.562
C20:0 Lact-Cer	0.04	0.73	-0.07	0.732	0.09	0.556
C20:1 Lact-Cer	-0.09	0.439	-0.14	0.485	-0.09	0.557
C22:0 Lact-Cer	0.04	0.722	-0.19	0.362	0.17	0.252
C22:1 Lact-Cer	-0.2	0.086	-0.36	0.074	-0.13	0.38
C24:0 Lact-Cer	0.08	0.482	-0.15	0.476	0.23	0.132
C24:1 Lact-Cer	-0.1	0.424	-0.27	0.188	0.09	0.545
C26:0 Lact-Cer	0.05	0.682	-0.26	0.207	0.22	0.137
C26:1 Lact-Cer	-0.2	0.095	-0.3	0.136	-0.11	0.484
Total Lact-Cer	-0.06	0.642	-0.26	0.192	0.11	0.482
**Hexosylceramide (Hex-Cer)**						
C14:0 Hex-Cer	-0.11	0.374	-0.17	0.406	-0.06	0.714
C16:0 Hex-Cer	0.08	0.496	-0.17	0.419	0.19	0.204
C18:0 Hex-Cer	0.14	0.228	0.01	0.969	0.19	0.201
C18:1 Hex-Cer	-0.05	0.702	0	0.985	-0.09	0.564
C20:0 Hex-Cer	0.03	0.771	-0.12	0.544	0.16	0.292
C20:1 Hex-Cer	0.01	0.952	0.16	0.428	-0.07	0.641
C22:0 Hex-Cer	0.11	0.338	-0.02	0.939	0.18	0.219
C22:1 Hex-Cer	-0.02	0.855	-0.03	0.877	-0.02	0.898
C24:0 Hex-Cer	0.15	0.199	0.03	0.877	0.22	0.141
C24:1 Hex-Cer	0.05	0.696	-0.09	0.657	0.11	0.465
C26:0 Hex-Cer	0.13	0.264	0.11	0.603	0.16	0.292
C26:1 Hex-Cer	0.05	0.671	-0.08	0.684	0.12	0.411
Total Hex-Cer	0.1	0.425	-0.13	0.528	0.2	0.19
**Dihydrosphingosine (dhSph)**	-0.11	0.34	0.09	0.67	-0.23	0.116
**Sphingosine**	-0.21	0.078	0.05	0.793	-0.33	***0*.*026***
**dhSph 1-phosphate**	-0.05	0.649	0.21	0.308	-0.11	0.469
**Sphingosine 1-phosphate (S1P)**	-0.11	0.358	0.15	0.458	-0.16	0.296
**C16:0 Cer : S1P Ratio**	0.21	0.074	-0.15	0.456	0.34	***0*.*023***
**C24:1 Cer : S1P Ratio**	0.2	0.088	-0.14	0.493	0.31	***0*.*039***

Italicized bold numbers indicate *p* < 0.05 (not corrected for multiple comparisons)

## Discussion

The current study examined the influence of race on blood sphingolipid profiles in SLE patients compared to healthy control subjects. The study cohort was from a well-characterized MUSC lupus population and their matched controls including both African-Americans and Whites. The targeted LC-MS/MS analyses revealed that African-American healthy control subjects had significantly higher plasma levels of almost all SM species and lower levels of Lact-Cer species compared to White control subjects. Levels of sphingosine and dhSph-1P were higher in African-American control subjects, but levels of most Cer species were not found to be different between African-American and White control subjects. Patients with SLE, irrespective of race, had higher plasma levels of Cer, and sphingoid bases (sphingosine and dhSph) and their phosphates (dhSph-1P and S1P) compared to control subjects. The ratios of C16:0 Cer/S1P and C24:1 Cer/S1P were however lower in SLE patients. In a lupus mouse model, we have previously also found that total plasma Cer and SIP levels were higher in the lupus mice compared to control mice [8]. We also found that C24:1 Cer levels were about 2 fold higher in the MRL/lpr lupus mice lacking the inducible nitric oxide synthase gene compared to their counterpart controls [8]. Using shotgun (MDMS-SL) (untargeted) lipidomics, Lu *et al*. [31] have recently reported that C24:1 Cer, the second abundant Cer species in human serum/plasma [8, 25], increased significantly (*p* < 0.05) in SLE patients compared to controls. Also in congruence with our data, Lu *et al*. demonstrated that levels of total SM and the majority of predominant SM species were not changed in SLE patients in comparison to control subjects.

Similar to the whole cohort data (SLE versus healthy control), White SLE patients exhibited higher levels of the sphingoid bases and their phosphates, and lower ratios of C16:0 Cer/S1P and C24:1 Cer/S1P, compared to White healthy subjects. African-American SLE patients on the other hand exhibited higher plasma levels of Cer species, Hex-Cer species, sphingosine and dhSph-1P compared to African-American control subjects. This difference in sphingolipid profile between African-American and White SLE patients compared to their respective healthy controls is worthy of future consideration.

Abnormal sphingolipid metabolism, due to mutations in genes encoding metabolizing enzymes’ expression and/or activity, is linked to the development of several genetic disorders such as Niemann-Pick disease, Fabry disease, Krabbe disease, Gaucher disease, and Tay-Sachs disease, some of which are also associated with increased risk of CVD. Hence, it is becoming increasingly clear that dysfunctional lipid metabolism and CVD extend far beyond cholesterol and triglycerides [10, 32, 33]. Despite the dyslipidemia and accelerated CVD associated with SLE [15], no studies have been conducted to determine whether levels of circulating sphingolipids in SLE patients may be associated with the occurrence of CVD. Since African-Americans are three times more likely than Whites to have lupus and develop severe symptoms (9), we investigated whether certain sphingolipid species can explain at least in part the race disparity in CVD manifestation in SLE. Among sphingolipids measured in our study, White SLE patients with atherosclerosis exhibited only lower levels of sphingoid bases compared to White SLE patients without atherosclerosis. On the contrary, African-American SLE patients with atherosclerosis had significantly higher levels of sphingoid bases, both sphingosine and dhSph, compared to African-American SLE patients without atherosclerosis. Data presented in Table 5 (last column) show that not only sphingoid bases (sphingosine and dhSph), but also C18:1 and C26:0 Lact-Cer, and C20:1 Hex-Cer levels may be dependent on race in SLE associated with CVD comorbidity. This significant interaction between race and atherosclerosis in SLE on the levels of these sphingolipids certainly indicates that there are several race-dependent factors, which may regulate the homeostasis of the sphingolipid metabolic and signaling pathways, including the activity of sphingolipid metabolizing enzymes, which may influence the levels of circulating sphingolipids. In another study, where the association of clinical and renal disease activity with circulating sphingolipids in patients with SLE have been investigated, the enzymes in the sphingolipid metabolism pathway were found to be altered in the SLE patients [20]. Ceramide synthase 5 and S1P lyase were upregulated, while glucosylceramidase and UDP-galactose ceramide galactosyltransferase were downregulated in SLE patients compared to controls [20]. Certainly, further ethnic physiological studies are needed to determine whether changes in sphingolipid metabolism in SLE patients could be associated with the development of CVD.

Notably, compared to African-American SLE patients without atherosclerosis, the African-American SLE patients with atherosclerosis had significantly higher plasma levels of SM, including most of SM species. In an epidemiological angiographic case-control study Jiang *et al*. showed that the plasma SM level is positively and independently correlated with age, body mass index, and systolic blood pressure [18]. They also showed lower mean plasma SM levels in men compared with women, in smokers compared with nonsmokers, and in Whites compared with other ethnic groups [18]. The same group then investigated whether plasma SM is an early atherogenic risk factor and examined the association between plasma SM level and carotid intimal-medial wall thickness, ankle-arm blood pressure index, and the Agatston coronary artery calcium score in asymptomatic adults [19]. They concluded that plasma SM is associated with subclinical atherosclerotic disease [19]. These reports are in agreement with our SM data in the African-American SLE cohort, with and without atherosclerosis. Interestingly, in our White SLE cohort, no difference in plasma SM was found between those who developed atherosclerosis and those who did not.

The levels of C24:1 Lact-Cer, the second dominant Lact-Cer next to C16 Lact-Cer in humans [25], were significantly lower in African-American SLE patients with atherosclerosis compared to those without. In contrast, levels of C18:1 Lact-Cer were significantly higher in African-American SLE patients with atherosclerosis compared to those without. The significance of alterations of Lact-Cer species levels in SLE patients with atherosclerosis remains to be determined.

When plasma sphingolipid profiles were compared between African-American and White SLE patients who developed atherosclerosis, we found that the African-American SLE patients with atherosclerosis had higher levels of the sphingoid bases, two Lact-Cer species (C18:0 and C20:0), and two Hex-Cer species (C20:1 and C22:1). This profile denotes a portion of the plasma sphingolipid profile identifying the whole African-American SLE cohort compared to that of the whole White SLE cohort (Table 3). The significance of this observation to the prediction, diagnosis, and/or prognosis of CVD in SLE remains to be determined.

Our data also revealed that the effect of SLE on three measures: plasma levels of C20:0 Hex-Cer, levels of dhSph-1P, and the C16:0 Cer/S1P Ratio could be dependent on race, African-Americans *versus* Whites. Furthermore, the effect of SLE associated with CVD comorbidity on the levels of C18:1 and C26:0 Lact-Cer, C20:1 Hex-Cer, and the sphingoid bases (sphingosine and dhSph) could also be dependent on race. Further ethnic studies in other SLE cohorts are necessary to verify this observation.

A cross-sectional study on a well-characterized European SLE cohort investigated the association of clinical and renal disease activity with circulating sphingolipids in female patients with SLE [20]. It was found that circulating plasma levels of Cer and Hex-Cer were increased, and levels of sphingosine and S1P were decreased in SLE patients compared to controls (all at p < 0.05). The ratio of C16:0 Cer/S1P was found to be the best indicator of SLE, and was also associated with active disease indices but not with accumulated damage due to the disease [20]. In our study, SLE patients, irrespective of race, had higher plasma levels of Cer, which is in agreement with the European study. However, contrary to the European study data, our data showed higher plasma levels of sphingoid bases (sphingosine and dhSph) and their phosphates (dhSph-1P and S1P), and lower C16:0 Cer/S1P ratio in SLE patients compared to control subjects. Not only the C16:0 Cer/S1P ratio but also the C24:1 Cer/S1P ratio was lower in SLE patients compared to controls in our study. A possible justification for this discrepancy, other than the probable race effect in our study, is the inclusion of the nephritis comorbidity in the European study but not in ours. It is possible that in the European study the plasma S1P fraction bound to albumin [26] is depleted due to its excretion with the urine (albuminuria). Lately, Patyna et al. showed that S1P and dhS1P levels were higher in plasma samples of SLE patients (irrespective of renal function) compared to healthy controls, which is in accord with data from our study [34]. However, in their study sphingosine and C16:0, C18:0, C20:0 and C24:1 Cer levels were elevated only in SLE patients suffering from impaired renal function, compared to healthy controls and SLE patients without impaired renal function [34].

In a small number (n = 7) of lupus nephritis patients, Nowling *et al*. found that levels of C16:0 Lact-Cer in the urine were significantly higher compared to lupus patients without nephritis or healthy control subjects (p < 0.001), and remained significantly higher after correcting for estimated glomerular filtration rate (eGFR) [6]. However, the authors found that serum levels of C16:0 Lact-Cer while exhibiting a trend towards higher levels in the lupus patients with nephritis compared to those without or the healthy controls, did not significantly differ among the groups (n = 10 each). Based on other pieces of data, the authors suggested that the elevated urine lipid is largely caused by renal-specific rather than systemic contributions [6]. We presume that the combination of blood and urine tests including sphingolipidomics, signs and symptoms of SLE, and findings from physical examination can provide insights into the underlying mechanism(s) leading to SLE and may uncover novel sphingolipid molecules as predictive/diagnostic/prognostic markers that identify individuals with lupus who are at increased risk for vascular disease. In fact, Checa *et al*. evaluated the effects of the immunosuppressive treatment on sphingolipid levels before and after treatment in 22 female SLE patients with active SLE and found that all plasma sphingolipids levels returned to ‘normal’ after the immunosuppressive treatment [20]. Patyna et al. found that plasma levels of C18:0 Cer but not C24:1 Cer were influenced by the glucocorticoid (prednisolone) treatment [34]. Notably, in the latter study correlation analyses revealed that elevated plasma ceramides (mainly C24:1 Cer) in patients with impaired renal function are not simply a reflection of kidney dysfunction as their levels did not correlate with renal markers (serum creatinine, eGFR, or albumin/creatinine ratio) [34]. In our study, plasma levels of sphingosine, C16:0 Cer/S1P ratio and C24:1 Cer/S1P ratio significantly correlated with SLEDAI in the African-American SLE patients, but there was no correlation between SLEDAI and plasma sphingolipid levels in the SLE cohort as a whole or in the White SLE patients. The lack of notable correlations between SLEDAI and plasma sphingolipid levels among Whites could be due to several factors 1) the fact that Whites with SLE tended to be healthier, 2) that sphingolipids are truly not associated with disease severity among Whites, and 3) that the number of Whites in our study was lower than the number of African Americans. Given the smaller sample size, we are hesitant to speculate about reasons for not observing any statistically significant associations.

Although this pstudyr highlights a number of associations involving sphingolipids, SLE status, and race/ethnicity, it should be emphasized that these findings are preliminary and mainly aimed at hypothesis generating. There were many statistical hypothesis tests conducted for this study, and even though we have identified those that remain statistically significant after controlling the false discovery rate, some of the findings we note may, in fact, be spurious. Validation of these findings in other cohorts would be ideal.

In conclusion, further longitudinal ethnic studies in more SLE cohorts are necessary to verify the use of sphingolipidomics as an additional tool to predict/diagnose SLE complications. The inclusion of ‘healthy’ relatives of SLE patients as an added control group in future studies will provide much needed information about the development of SLE and its comorbidities. In addition, the inclusion of patients who do not have SLE but have kidney disease (e.g., diabetic nephropathy) or CVD in future studies as control groups would validate the specificity of plasma sphigolipdomics profiling compared to other diagnostic/prognostic assays. Knowing that the modulation of the sphingolipid pathway is presently used as a therapeutic intervention in autoimmune diseases such as multiple sclerosis [35] and psoriasis [36], we believe that sphingolipid metabolizing enzymes could be targeted for future development of therapies to tailor treatments given to SLE patients and to prevent acceleration of vascular comorbidities in high-risk patients including African Americans. In future epidemiological studies, it will be extremely important to establish the intra-individual changes in sphingolipids in relation to disease onset and progression. Longitudinal studies are warranted to proof the potential sphingolipid ‘markers’ for early diagnosis of SLE comorbidities, including atherosclerosis, not as a substitute or an alternative, but rather as an augmentation of the traditional diagnostic markers. It has been recently speculated that sphingolipids may be the new markers for metabolic and cardiovascular diseases similar to HDL and LDL cholesterol [37, 38]. In fact, Mayo Clinic Laboratories have started performing a diagnostic test that quantifies plasma levels of ceramides to identify patients at higher risk of developing major adverse cardiovascular events.

## Supporting information

S1 Fig Demographics of the SLE registry population. (TIF)

S1 File Data set. (CSV)

S2 File Code book. (XLSX)
