# Supplementary figures and images for: Race disparity in blood sphingolipidomics associated with lupus cardiovascular comorbidity
Source: PLoS One. 2019 Nov 20;14(11):e0224496. doi: 10.1371/journal.pone.0224496 (PMC6867606; doi:10.1371/journal.pone.0224496)

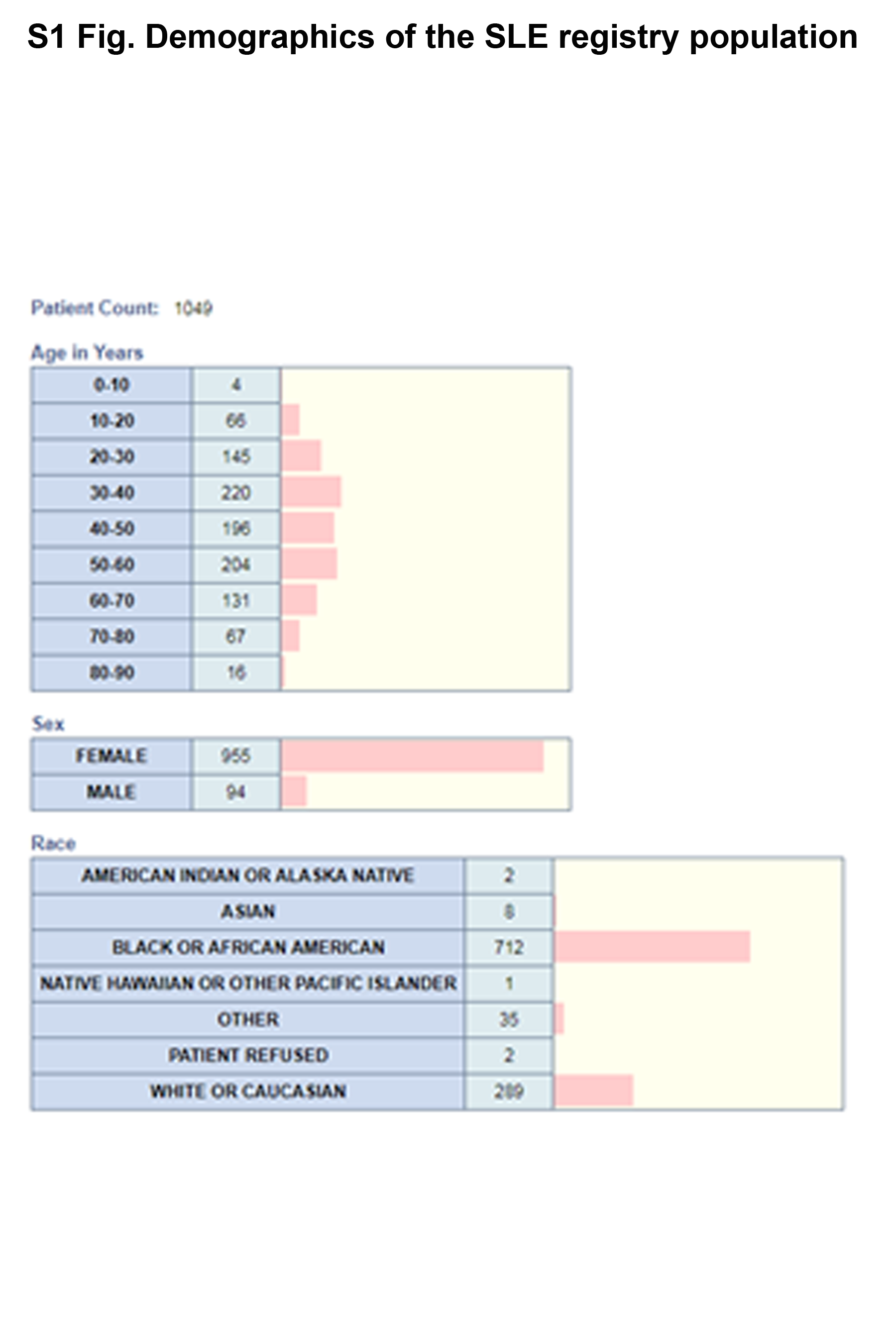

Supplement: S1 Fig — (TIF) [file pone.0224496.s001.tif]
